# Theoretical Investigation of Gamma- and Neutron-Shielding Properties of Polysulfone (PSU) Polymer Material Using Geant4

**DOI:** 10.3390/polym14163374

**Published:** 2022-08-18

**Authors:** Hanan Akhdar

**Affiliations:** Department of Physics, Faculty of Science, Imam Mohammad Ibn Saud Islamic University (IMSIU), P.O. Box 90950, Riyadh 11623, Saudi Arabia; hfakdar@imamu.edu.sa

**Keywords:** polymer, PSU, Geant4, gamma, neutron, attenuation

## Abstract

Polymers are widely used materials that have many medical and industrial applications. Some polymers have even been introduced as radiation-shielding materials; therefore, many studies are focusing on new polymers and their interactions with photons and neutrons. Research has focused on theoretical estimation of the shielding effectiveness of different materials. It is well known that theoretical studies on the shielding properties of different materials through modeling and simulation have many benefits, as they help scientists to choose the right shielding material for a specific application, and they are also much more cost-effective and take much less time compared to experimental studies. In this study, polysulfone (PSU) was investigated. PSU is a high-temperature, amber-colored, semi-transparent plastic material with good mechanical properties. It is resistant to degradation from hot water and steam and is often used in medical and food preparation applications, where repeated sterilization is required. The interactions of photons and neutrons with PSU were investigated using a Monte Carlo-based simulation toolkit, Geant4, within a wide range of energies of both photons and neutrons. The mass attenuation coefficients (*µ_m_*), the half-value layers (*HVL*), the effective atomic numbers (*Z_eff_*), and the effective electron densities (*N_eff_*) of gammas were investigated. In addition, the effective removal cross-sections (Σ*_R_*) and the mean free paths (*λ*) of neutrons were also studied. The results were then compared to other commonly used polymer materials.

## 1. Introduction

The properties of polymers have made them very good candidates for many applications, as they are affordable and easy to shape and handle. Recently, researchers have studied the gamma- and neutron-shielding properties of different polymers, in order to evaluate their ability to attenuate radiation, especially those with certain uses in radiation-related utilities [1,2,3].

Many of these studies showed that the gamma attenuation performances of polymers are promising, and the ability of polymers to attenuate gamma rays may be increased by adding different composites with high atomic numbers to these polymers [1,2,3,4,5]. 

In addition, many studied polymers have high thermal neutron cross-sections and high sensitivities to neutron particles, which make them good neutron-shielding materials as well [6,7,8]. The shielding of neutrons and gamma rays is always a major concern, because any material that effectively attenuates neutrons and gammas, attenuates all other types of radiation effectively. 

Polysulfone (PSU) is a relatively newly introduced polymer with promising properties that have made it attractive to be focused on by researchers. PSU’s photon shielding capability was studied and compared to other polymers using Monte Carlo simulation software at certain energies [9]. 

In this work, polysulfone (PSU) was investigated theoretically. The gamma- and neutron-shielding properties of PSU were both studied theoretically using the Monte Carlo simulation toolkit Geant4 at a wide energy range, between 0.1 and 20 MeVs. The obtained results were validated using EpiXS and WinNC software. Finally, the performance of PSU as a shielding material against gammas and neutrons was compared to other common polymers, where the gamma mass attenuation coefficients and the neutron effective removal cross-sections were investigated in the same energy range as used with Geant4. 

## 2. Polysulfone

**Figure 1:**
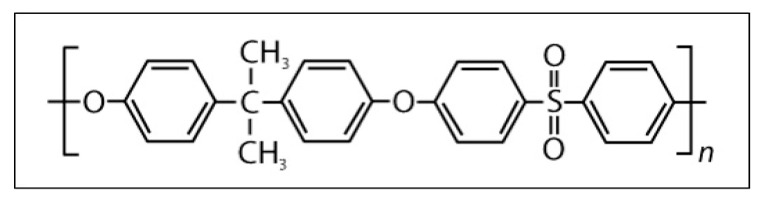
PSU repeating unit.

Polysulfone (PSU) is a high-temperature rigid plastic with high mechanical strength. It is remarkably strong over a wide range of temperatures and has good resistance to hydrolysis, excellent stability, and good chemical compatibility. It has a density between 1.24 and 1.25 g/cm^3^ and is a repeating unit, as shown in Figure 1 [10,11,12,13,14]. Table 1 summarizes the PSU element fractions.

## 3. Theory

### 3.1. Gamma Attenuation

The gamma mass attenuation coefficient (*μ_m_*) can be calculated using Equation (1) [15]:

(1) $$I=I_{0}e^{-\mu_{m}x}$$

where (*I*_0_) is the incident intensity of photons, and (*I*) is the attenuated photons’ intensity after passing through a mass per unit area (*x*) layer of material. The mass attenuation coefficient can be used to calculate the linear attenuation coefficient (*μ*) using Equation (2):

(2) $$\mu =\mu_{m}\rho$$

where (*ρ*) is the density of the material [15,16]. The linear attenuation coefficient is used to determine the half-value layer (*HVL*) of the material, which is a very important property of any shielding material and can be found using Equation (3) [16,17]:

(3) $$HVL=\frac{\ln2}{\mu }$$

The total atomic cross-section can be calculated using Equation (4) [18]:

(4) $$\sigma_{t.a}=\frac{\mu_{m}}{N_{A}\sum_{i}^{n}\left(w_{i}/A_{i}\right)}$$

where (*N_A_*) is Avogadro’s number, and (*A_i_*) is the atomic weight of an element of the compound, while the total electronic cross-section for the element is given by Equation (5) [18]:

(5) $$\sigma_{t.el}=\frac{1}{N_{A}}\sum_{i}^{n}\frac{f_{i}A_{i}}{Z_{i}}{\left(\mu_{mt}\right)}_{i}$$

where (*f_i_*) is the number of atoms of the element (*i*) relative to the total number of atoms of all elements in the compound, and (*Z_i_*) is the atomic number of the *i*th element in the compound. The effective atomic number (*Z_eff_*) of the compound can be found from the ratio between the total atomic cross-section and the total electronic cross-section using Equation (6) [16,17]:

(6) $$Z_{eff}=\frac{\sigma_{t,a}}{\sigma_{t,el}}$$

The effective electron density is given by Equation (7) [16,17]:

(7) $$N_{eff}=\frac{\mu_{m}}{\sigma_{t,el}}$$

These parameters are all important when studying the gamma-shielding properties of any material and were all investigated theoretically in this work on polysulfone polymers.

### 3.2. Neutron Attenuation

Neutron attenuation is described by the neutron removing cross-section (Σ*_R_*), which is the probability of neutron reactions within a material, and is given by Equation (8) [19]:

(8) $$\Sigma_{R}={\sum_{i}\rho_{i}\left(\Sigma_{R}/\rho \right)}_{i}$$

where (*ρ_i_*) is the partial density, and (*Σ_R_/ρ*) is the mass removal cross-section, which can be calculated for any compound using Equation (9) [20]:

(9) ΣRρ=0.206A−13Z−0.294

where (*A*) is the atomic weight, and (*Z*) is the atomic number.

The fast neutron removal cross-section of any element can be calculated using Equations (10) and (11) [21]:

(10) $$\Sigma_{R}=0.190Z^{-0.743}\;\mathrm{if}\;Z\;\leq \;8$$

(11) $$\Sigma_{R}=0.125Z^{-0.565}\;\mathrm{if}\;Z\;>\;8$$

The mean free Path (*λ*), which is the distance that the neutron travels without interaction, is given by Equation (12) [19,20]:

(12) $$\lambda =\frac{1}{\Sigma_{R}}$$

The (*HVL*), which is the thickness needed to reduce the neutron intensity to half of its original value, is given by Equation (13) [19,20]:

(13) $$HVL=\frac{\ln2}{\Sigma_{R}}$$

## 4. Methods

**Figure 2:**
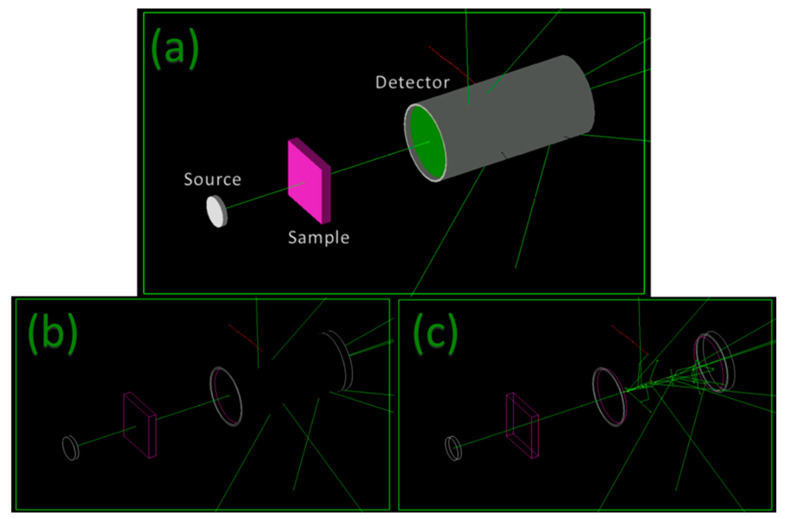
Geant4 visualization of the developed code. (**a**) Solid surface view, (**b**) wire frame view, (**c**) hidden edges view.

In this work, a very well-known Monte Carlo-based toolkit, Geant4, which is utilized in nuclear physics, nuclear engineering, and medical physics, was used to evaluate the gamma- and neutron-shielding properties of PSU. A Geant4 code was developed to study the interactions of both gammas and neutrons in the energy range between 0.1 and 20 MeVs. A source was placed in front of a sample made of PSU and shot mono-energetic gamma and neutron particles in the direction of the sample, followed by a detector. The attenuation of both gammas and neutrons was measured by determining the ratio between the number of particles reaching the detector with and without the sample [22]. For each unit of energy, 1,000,000 mono-energetic particles were emitted in the direction perpendicular to the sample. Figure 2 shows a screenshot of the Geant4 simulation code used in this study. The gamma-shielding-related results were compared to those obtained from EpiXS, a Windows-based application based on EPICS2017 of ENDF/B-VIII and EPDL97 of ENDF/B-VI.8 and which is a user-friendly software constructed for photon attenuation, dosimetry, and shielding. It performs data library interpolation between 1 keV and 100 GeV and calculates partial or total cross-sections, as well as mass and linear attenuation coefficients for any user-defined material [23]. On the other hand, the neutron shielding results obtained from Geant4 were compared with those from WinNC at certain energies. WinNC is a user-friendly Windows-based platform that provides the neutron attenuation coefficients of any material [20].

## 5. Results

### 5.1. Gamma Shielding Properties of PSU

**Figure 3:**
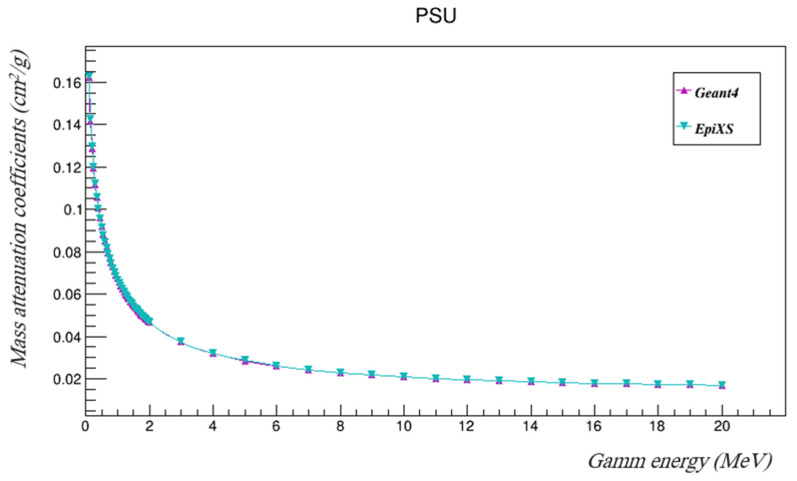
The mass attenuation coefficients of PSU at the investigated gamma energies using both Geant4 and EpiXS.

The gamma mass attenuation coefficients at the studied energies were found using Geant4. In order to validate the results, they were compared to those from EpiXS, which is a Windows-based program for photon attenuation, dosimetry, and shielding, based on the EPICS2017 and EPDL9 databases, and which allows obtaining photon cross-section data for any sample [24]. Root (6.10/04) software was used to plot the mass attenuation coefficients found using both Geant4 and EpiXS in the studied energy range, as shown in Figure 3 [23]. Table 2 tabulates the results and the percentage differences between the mass attenuation coefficients found using Geant4 and EpiXS, as calculated by Equation (14).

(14) $$\%\;\Delta =100*\left(\mu_{EpiXS}-\mu_{G4}\right)/\mu_{EpiXS}$$

**Figure 4:**
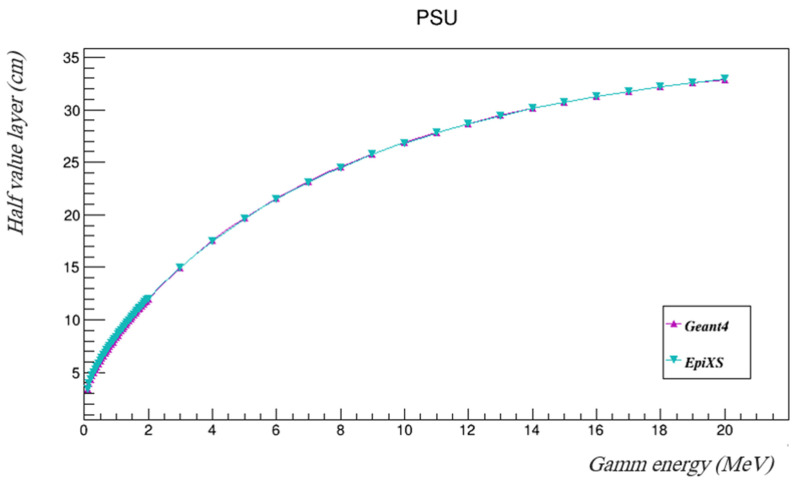
The half-value layers of PSU in the investigated gamma energies using both Geant4 and EpiXS.

The results of Geant4 agree very well with those found using EpiXS, where the difference between them was found to be less than 1%. The half-value layers of PSU were estimated using both Geant4 and EpiXS, as listed in Table 3 and illustrated in Figure 4.

**Figure 5:**
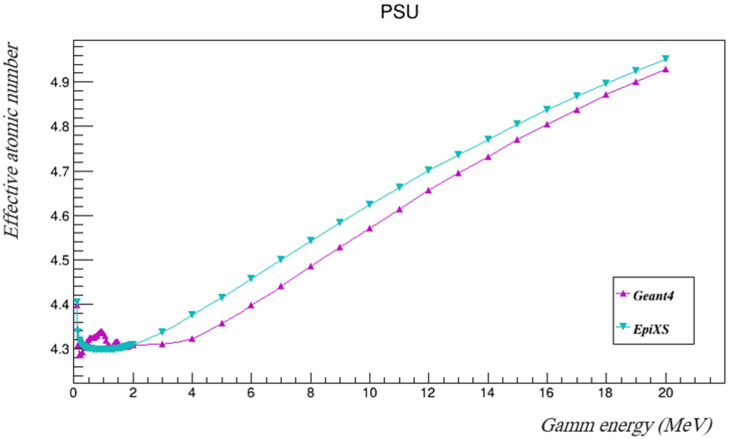
The effective atomic numbers of PSU with the investigated gamma energies using both Geant4 and EpiXS.

**Figure 6:**
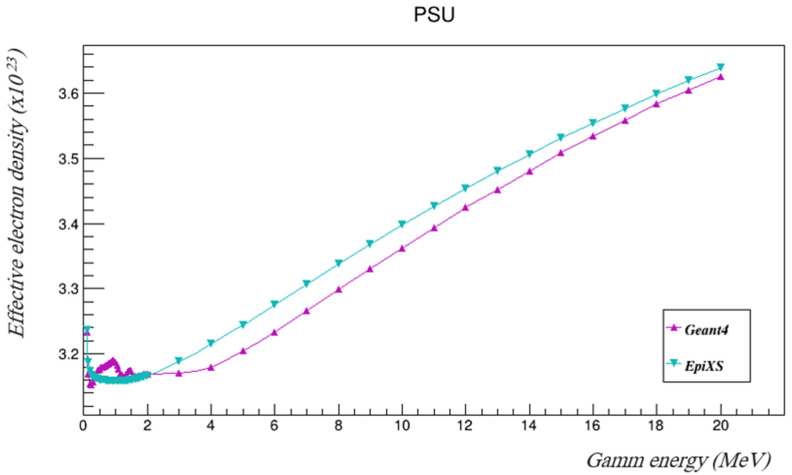
The effective electron densities of PSU with the investigated gamma energies using both Geant4 and EpiXS.

The mass attenuation coefficients found using Geant4 were then used to calculate the effective atomic numbers and the effective electron densities using Equations (6) and (7), and then they were compared to those found using EpiXS. Table 4 and Table 5 summarize the results and Figure 5 and Figure 6 show them plotted.

The results show that the effective atomic numbers of PSU fell within the range of the atomic numbers of its components. Some fluctuations of both manually calculated Z_eff_ and N_eff_ (based on the attenuations found by Geant4) were seen at lower energies, but the differences between the calculated values using the mass attenuation coefficients found by Geant4 and the tabulated values estimated by EpiXS are still very acceptable, as they are less than 1.3%. The values of *Z_eff_* and *N_eff_* started to agree more at higher energies.

### 5.2. Neutron Shielding Properties of PSU

**Figure 7:**
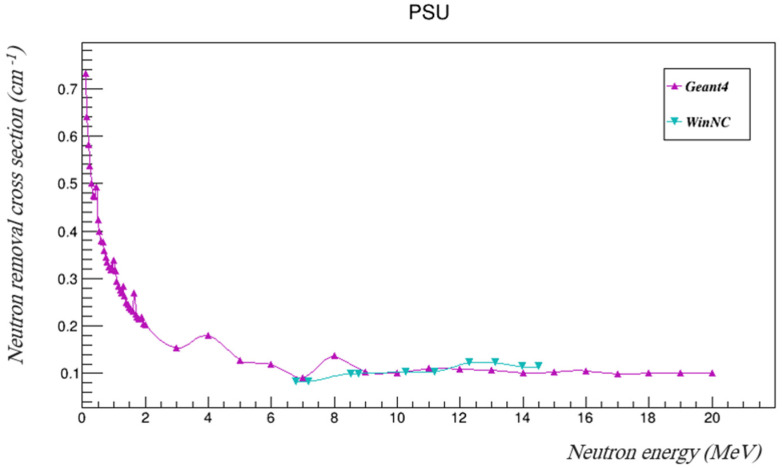
The neutron removal cross-sections of PSU at the investigated gamma energies using both Geant4 and WinNC.

**Figure 8:**
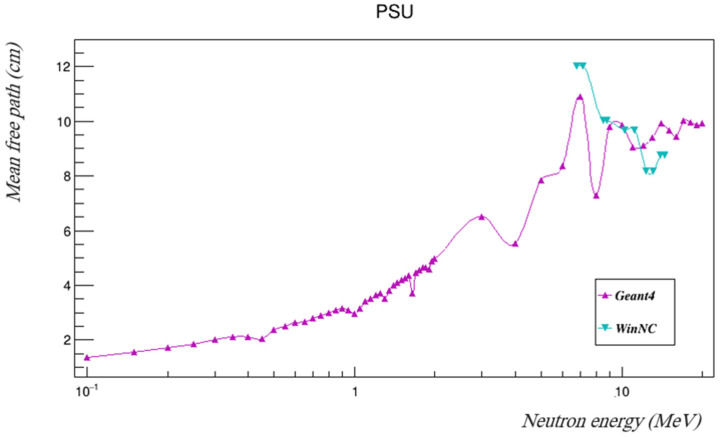
The neutron mean free paths of PSU at the investigated gamma energies using both Geant4 and WinNC.

The neutron removal cross-sections of PSU were found using Geant4. In order to validate the results, they were compared to removal cross-sections measured using the WinNC toolkit, which is a database used to estimate the neutron removal cross-sections of materials [19]. Table 6 summarizes the removal cross-sections and mean free paths as found using Geant4 in the investigated energy range, and those found using WinNC at the available energy intervals are tabulated in Table 7. The removal cross-sections found using both Geant4 and WinNC are plotted in Figure 7, and the mean free paths are shown in Figure 8.

The results of WinNC are comparable to those of Geant4 at the available energies, as can be seen in Figure 7 and Figure 8. 

## 6. Comparison with Other Commonly Used Polymers

**Figure 9:**
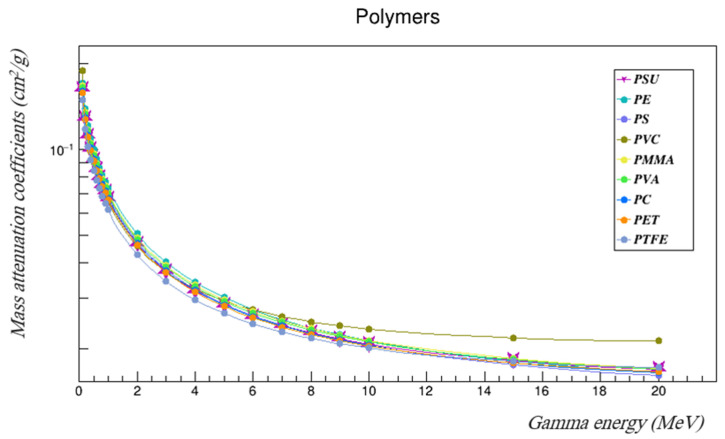
The gamma mass attenuation coefficients of the polymers compared to PSU in the studied energy range.

**Figure 10:**
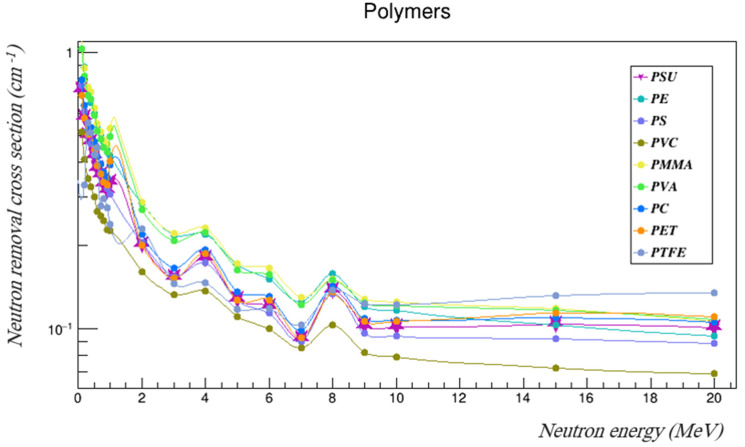
The neutron removal cross-sections of the polymers compared to PSU at the studied energy range.

The gamma mass attenuation coefficients and effective neutron removal cross-sections of commonly used polymers were investigated using Geant4 and compared with those of the PSU [25]. The compared polymers were polyethylene (PE), polystyrene (PS), polyvinyl chloride (PVC), polymethyl methacrylate (PMMA), polyvinyl alcohol (PVA), polycarbonate (PC), polyethylene terephthalate (PET), and polytetrafluoroethylene (PTFE). Table 8 summarizes the properties of those polymers. Table 9 and Table 10 tabulate the results, and Figure 9 and Figure 10 represent the gamma mass attenuation coefficients and neutron removal cross-sections of all compared polymers. 

In addition, the fast neutron removal cross-sections were calculated and compared for all the studied polymers using Equations (8), (10), and (11), with the use of the fast neutron removal cross-sections and the weight fractions of each element in each polymer [26,27,28]. Table 11 lists the calculations of the fast neutron removal cross-sections.

The results show that the gamma- and neutron-shielding properties of PSU fell within the range of the properties of the other commonly used polymers. It had higher gamma attenuation coefficients than PET and PTFE across the entire studied energy range, and it had higher coefficients than PVC at energies between 0.3 and 3 MeV. It also had better gamma-shielding properties than PS and PC at energies higher than 6 MeVs. PSU’s neutron-shielding properties were better than those of PVC across the entire investigated energy range, and it had higher neutron removal cross-sections than PS at energies higher than 4 MeV, which were also better than those of PET and PTFE at some neutron energies. The fast neutron removal cross-section of PSU, which agrees with that found using WinNC, was higher than those of PE, PS, and PVC, and almost equal to that of PC. These results indicate that PSU is a good shielding candidate, and further studies of PSU performance as a gamma- and neutron-shielding material are needed. Doping or adding nano-particle composites to PSU could enhance its shielding properties even more, which should be considered in future studies.

## 7. Conclusions

The gamma- and neutron-shielding properties of PSU were studied at the energy range between 0.1 and 20 MeV. The resulting data provided in this study are important for the selection of PSU polymers when used in particular fields or applications.

The gamma characteristics of PSU were investigated utilizing the mass attenuation coefficients, half-value layers, effective atomic numbers, and electron effective densities, to show the ability of PSU to act as a gamma-shielding material. The results obtained from Geant4 were validated using the results from EpiXS, and the agreement between them was excellent, as the percentage difference was less than 1% over the complete investigated energy range. 

The neutron properties of PSU were investigated, as well using the neutron removal cross-sections and mean free paths. The Geant4 results were compared to the WinNC results at certain available neutron energy intervals and they showed a good agreement.

PSU’s performance as a gamma- and neutron-shielding material was also compared to other common polymer behaviors. These comparison results confirmed that PSU can be considered a good shielding material, as the gamma- and neutron-shielding properties of PSU were comparable to, and in some cases higher than, those of other compared polymers. 

Based on the results of the current study, PSU is a very good candidate to be considered as a gamma- and neutron-shielding material, and the computed gamma mass attenuation coefficients and neutron removal cross-sections will be helpful during the selection of a shielding materials and for calculating other radiation-related parameters. Further experimental studies could validate these results at different gamma or neutron energies. Different techniques of manufacturing PSU may have effects on its shielding properties, and adding composites may also enhance its effectiveness in shielding gamma rays and neutrons, which will need more focused future research. 

## Figures and Tables

**Table 1:** PSU elements.

Element	Atom Fraction	Weight Fraction
**H (Hydrogen)**	0.407407	0.050109
**C (Carbon)**	0.500000	0.732814
**O (Oxygen)**	0.074074	0.144619
**S (Sulfur)**	0.018519	0.072458

**Table 2:** The mass attenuation coefficients of PSU in the investigated gamma energy range.

Gamma Energy (MeV)	Mass Attenuation Coefficient (cm^2^/g)		% Δ	Gamma Energy (MeV)	Mass Attenuation Coefficient (cm^2^/g)		% Δ
	EpiXS	Geant4			EpiXS	Geant4	
**0.1**	0.162728	0.162476	0.15%	1.55	0.053361	0.053418	−0.11%
**0.15**	0.142513	0.14162	0.63%	1.6	0.052463	0.052505	−0.08%
**0.2**	0.129550	0.12866	0.69%	1.65	0.051606	0.051642	−0.07%
**0.25**	0.119812	0.119206	0.51%	1.7	0.050790	0.050823	−0.07%
**0.3**	0.112078	0.111754	0.29%	1.75	0.050028	0.050046	−0.04%
**0.35**	0.105609	0.105611	0.00%	1.8	0.049268	0.049304	−0.07%
**0.4**	0.100227	0.100394	−0.17%	1.85	0.048547	0.048595	−0.10%
**0.45**	0.095497	0.095862	−0.38%	1.9	0.047888	0.047915	−0.06%
**0.5**	0.091446	0.091874	−0.47%	1.95	0.047230	0.047254	−0.05%
**0.55**	0.087799	0.088304	−0.58%	2	0.046577	0.046605	−0.06%
**0.6**	0.084555	0.085079	−0.62%	3	0.037400	0.03728	0.32%
**0.65**	0.081605	0.08216	−0.68%	4	0.032038	0.031913	0.39%
**0.7**	0.078928	0.079508	−0.73%	5	0.028524	0.028413	0.39%
**0.75**	0.076487	0.077087	−0.78%	6	0.026052	0.025958	0.36%
**0.8**	0.074235	0.074862	−0.84%	7	0.024214	0.024146	0.28%
**0.85**	0.072123	0.072795	−0.93%	8	0.022825	0.022759	0.29%
**0.9**	0.070191	0.070852	−0.94%	9	0.021722	0.021669	0.24%
**0.95**	0.068411	0.068996	−0.86%	10	0.020833	0.020791	0.20%
**1**	0.066712	0.067192	−0.72%	11	0.020099	0.020075	0.12%
**1.05**	0.065103	0.065422	−0.49%	12	0.019501	0.019481	0.10%
**1.1**	0.063630	0.063813	−0.29%	13	0.018995	0.018981	0.07%
**1.15**	0.062190	0.062342	−0.24%	14	0.018557	0.018558	−0.01%
**1.2**	0.060911	0.060995	−0.14%	15	0.018191	0.018196	−0.03%
**1.25**	0.059652	0.059754	−0.17%	16	0.017879	0.017885	−0.04%
**1.3**	0.058454	0.058607	−0.26%	17	0.017594	0.017615	−0.12%
**1.35**	0.057313	0.057542	−0.40%	18	0.017366	0.01738	−0.08%
**1.4**	0.056258	0.056513	−0.45%	19	0.017151	0.017174	−0.13%
**1.45**	0.055206	0.055415	−0.38%	20	0.016972	0.016993	−0.12%
**1.5**	0.054278	0.054385	−0.20%				

**Table 3:** The half-value layer of PSU in the investigated energy range.

Gamma Energy (MeV)	HVL (cm)		% Δ	Gamma Energy (MeV)	HVL (cm)		% Δ
	EpiXS	Geant4			EpiXS	Geant4	
**0.1**	3.435110	3.440445	−0.16%	1.55	10.475600	10.464542	0.11%
**0.15**	3.922360	3.947110	−0.63%	1.6	10.654900	10.646449	0.08%
**0.2**	4.314850	4.344704	−0.69%	1.65	10.831900	10.824385	0.07%
**0.25**	4.665560	4.689275	−0.51%	1.7	11.006000	10.998667	0.07%
**0.3**	4.987480	5.001966	−0.29%	1.75	11.173600	11.169629	0.04%
**0.35**	5.292990	5.292911	0.00%	1.8	11.345900	11.337636	0.07%
**0.4**	5.577210	5.567959	0.17%	1.85	11.514400	11.503052	0.10%
**0.45**	5.853500	5.831197	0.38%	1.9	11.672900	11.666228	0.06%
**0.5**	6.112790	6.084321	0.47%	1.95	11.835400	11.829493	0.05%
**0.55**	6.366720	6.330265	0.57%	2	12.001400	11.994251	0.06%
**0.6**	6.610950	6.570227	0.62%	3	14.946300	14.994318	−0.32%
**0.65**	6.849930	6.803713	0.67%	4	17.448000	17.515829	−0.39%
**0.7**	7.082300	7.030627	0.73%	5	19.597400	19.673452	−0.39%
**0.75**	7.308270	7.251385	0.78%	6	21.457000	21.534389	−0.36%
**0.8**	7.529990	7.466965	0.84%	7	23.085700	23.150884	−0.28%
**0.85**	7.750540	7.678936	0.92%	8	24.490400	24.560826	−0.29%
**0.9**	7.963880	7.889551	0.93%	9	25.734300	25.796983	−0.24%
**0.95**	8.171060	8.101757	0.85%	10	26.832400	26.885619	−0.20%
**1**	8.379210	8.319239	0.72%	11	27.811700	27.845203	−0.12%
**1.05**	8.586220	8.544381	0.49%	12	28.665000	28.694831	−0.10%
**1.1**	8.785010	8.759850	0.29%	13	29.428600	29.449648	−0.07%
**1.15**	8.988420	8.966559	0.24%	14	30.123200	30.121386	0.01%
**1.2**	9.177160	9.164561	0.14%	15	30.729700	30.719794	0.03%
**1.25**	9.370870	9.354834	0.17%	16	31.265600	31.254314	0.04%
**1.3**	9.562860	9.538015	0.26%	17	31.771100	31.733190	0.12%
**1.35**	9.753250	9.714497	0.40%	18	32.189200	32.162996	0.08%
**1.4**	9.936120	9.891381	0.45%	19	32.591500	32.548979	0.13%
**1.45**	10.125600	10.087389	0.38%	20	32.936400	32.895677	0.12%
**1.5**	10.298700	10.278323	0.20%				

**Table 4:** The effective atomic numbers of PSU in the investigated energy range.

Gamma Energy (MeV)	*Z_eff_*		% Δ	Gamma Energy (MeV)	Z_eff_		% Δ
	EpiXS	Geant4			EpiXS	Geant4	
**0.1**	4.404337	4.396582	0.18%	1.55	4.300502	4.307547	−0.16%
**0.15**	4.337329	4.309241	0.65%	1.6	4.301165	4.304129	−0.07%
**0.2**	4.318226	4.287654	0.71%	1.65	4.301902	4.303667	−0.04%
**0.25**	4.310152	4.287462	0.53%	1.7	4.302711	4.303989	−0.03%
**0.3**	4.306020	4.292653	0.31%	1.75	4.303560	4.304683	−0.03%
**0.35**	4.303653	4.302832	0.02%	1.8	4.304492	4.304204	0.01%
**0.4**	4.302168	4.308416	−0.15%	1.85	4.305450	4.306750	−0.03%
**0.45**	4.301142	4.316691	−0.36%	1.9	4.306484	4.308801	−0.05%
**0.5**	4.300469	4.319699	−0.45%	1.95	4.307568	4.308039	−0.01%
**0.55**	4.299894	4.323750	−0.55%	2	4.308717	4.308834	0.00%
**0.6**	4.299378	4.323750	−0.57%	3	4.337838	4.310388	0.63%
**0.65**	4.299079	4.325123	−0.61%	4	4.374776	4.323069	1.18%
**0.7**	4.298809	4.327383	−0.66%	5	4.415080	4.356947	1.32%
**0.75**	4.298622	4.329494	−0.72%	6	4.457275	4.397131	1.35%
**0.8**	4.298483	4.331436	−0.77%	7	4.499886	4.440384	1.32%
**0.85**	4.298325	4.333856	−0.83%	8	4.541972	4.486364	1.22%
**0.9**	4.298194	4.337504	−0.91%	9	4.583460	4.528093	1.21%
**0.95**	4.298091	4.337786	−0.92%	10	4.623780	4.571470	1.13%
**1**	4.297999	4.333954	−0.84%	11	4.662772	4.613785	1.05%
**1.05**	4.297914	4.328076	−0.70%	12	4.700105	4.656332	0.93%
**1.1**	4.297849	4.318061	−0.47%	13	4.736266	4.694398	0.88%
**1.15**	4.297852	4.309302	−0.27%	14	4.771205	4.732073	0.82%
**1.2**	4.297913	4.307433	−0.22%	15	4.804448	4.770685	0.70%
**1.25**	4.298060	4.302927	−0.11%	16	4.836548	4.805191	0.65%
**1.3**	4.298273	4.304524	−0.15%	17	4.867063	4.837510	0.61%
**1.35**	4.298564	4.308577	−0.23%	18	4.896777	4.872082	0.50%
**1.4**	4.298939	4.314807	−0.37%	19	4.924794	4.899979	0.50%
**1.45**	4.299388	4.317494	−0.42%	20	4.952303	4.930453	0.44%
**1.5**	4.299912	4.314762	−0.35%				

**Table 5:** The electron effective densities of PSU in the investigated energy range.

Gamma Energy (MeV)	*N_eff_* (×10^23^ e^−^/g)		% Δ	Gamma Energy (MeV)	*N_eff_* (×10^23^ e^−^/g)		% Δ
	EpiXS	Geant4			EpiXS	Geant4	
**0.1**	3.236574	3.233453	0.10%	1.55	3.160269	3.167973	−0.24%
**0.15**	3.187332	3.169219	0.57%	1.6	3.160757	3.165460	−0.15%
**0.2**	3.173294	3.153342	0.63%	1.65	3.161298	3.165119	−0.12%
**0.25**	3.167361	3.153201	0.45%	1.7	3.161892	3.165357	−0.11%
**0.3**	3.164324	3.157019	0.23%	1.75	3.162516	3.165867	−0.11%
**0.35**	3.162585	3.164505	−0.06%	1.8	3.163201	3.165514	−0.07%
**0.4**	3.161494	3.168612	−0.23%	1.85	3.163905	3.167387	−0.11%
**0.45**	3.160740	3.174698	−0.44%	1.9	3.164665	3.168895	−0.13%
**0.5**	3.160245	3.176910	−0.53%	1.95	3.165462	3.168335	−0.09%
**0.55**	3.159823	3.179889	−0.64%	2	3.166306	3.168919	−0.08%
**0.6**	3.159443	3.179889	−0.65%	3	3.187706	3.170063	0.55%
**0.65**	3.159223	3.180899	−0.69%	4	3.214850	3.179389	1.10%
**0.7**	3.159025	3.182561	−0.75%	5	3.244468	3.204304	1.24%
**0.75**	3.158888	3.184114	−0.80%	6	3.275476	3.233857	1.27%
**0.8**	3.158786	3.185542	−0.85%	7	3.306789	3.265668	1.24%
**0.85**	3.158669	3.187322	−0.91%	8	3.337716	3.299483	1.15%
**0.9**	3.158573	3.190005	−1.00%	9	3.368204	3.330173	1.13%
**0.95**	3.158497	3.190212	−1.00%	10	3.397833	3.362074	1.05%
**1**	3.158430	3.187394	−0.92%	11	3.426487	3.393194	0.97%
**1.05**	3.158367	3.183071	−0.78%	12	3.453922	3.424486	0.85%
**1.1**	3.158320	3.175705	−0.55%	13	3.480495	3.452481	0.80%
**1.15**	3.158322	3.169264	−0.35%	14	3.506170	3.480190	0.74%
**1.2**	3.158366	3.167889	−0.30%	15	3.530599	3.508586	0.62%
**1.25**	3.158475	3.164575	−0.19%	16	3.554188	3.533963	0.57%
**1.3**	3.158631	3.165750	−0.23%	17	3.576612	3.557733	0.53%
**1.35**	3.158845	3.168731	−0.31%	18	3.598448	3.583158	0.42%
**1.4**	3.159121	3.173312	−0.45%	19	3.619037	3.603676	0.42%
**1.45**	3.159450	3.175288	−0.50%	20	3.639252	3.626087	0.36%
**1.5**	3.159836	3.173279	−0.43%				

**Table 6:** The neutron removal cross-sections and mean free paths of PSU in the investigated energy range as found using Geant4.

Neutron Energy (MeV)	Σ*_R_* (cm^−1^)	λ (cm)	Neutron Energy (MeV)	Σ*_R_* (cm^−1^)	λ (cm)
	Geant4			Geant4	
0.1	0.73269	1.36483	1.55	0.23447	4.26494
0.15	0.64015	1.56213	1.6	0.23018	4.34443
0.2	0.58089	1.7215	1.65	0.26957	3.70961
0.25	0.5377	1.85977	1.7	0.22408	4.46269
0.3	0.50044	1.99824	1.75	0.2193	4.55996
0.35	0.47305	2.11394	1.8	0.21532	4.64425
0.4	0.47298	2.11425	1.85	0.21457	4.66048
0.45	0.49241	2.03083	1.9	0.21771	4.59327
0.5	0.42252	2.36675	1.95	0.20489	4.88067
0.55	0.3983	2.51067	2	0.20128	4.9682
0.6	0.37859	2.64138	3	0.15341	6.51848
0.65	0.37678	2.65407	4	0.18038	5.54385
0.7	0.35914	2.78443	5	0.12731	7.85484
0.75	0.34466	2.90141	6	0.11962	8.35981
0.8	0.33313	3.00183	7	0.091677	10.90786
0.85	0.32386	3.08775	8	0.13744	7.2759
0.9	0.31698	3.15477	9	0.10202	9.802
0.95	0.32408	3.08566	10	0.10128	9.87362
1	0.33818	2.95701	11	0.11052	9.04814
1.05	0.31679	3.15667	12	0.10985	9.10332
1.1	0.29444	3.39628	13	0.10649	9.39055
1.15	0.28363	3.52572	14	0.10072	9.92851
1.2	0.27505	3.6357	15	0.10356	9.65624
1.25	0.26932	3.71306	16	0.10587	9.44555
1.3	0.28402	3.52088	17	0.099761	10.02396
1.35	0.26264	3.80749	18	0.10028	9.97208
1.4	0.24946	4.00866	19	0.10146	9.8561
1.45	0.24388	4.10038	20	0.10075	9.92556
1.5	0.23863	4.19059			

**Table 7:** The neutron removal cross-sections and mean free paths of PSU using WinNC.

Neutron Energy (MeV)	Σ*_R_* (cm^−1^)	λ (cm)
	WinNC	
**Thermal**	1.502065	0.66575
**6.8–7.17**	0.083304	12.00423
**8.54–8.77**	0.099720	10.02808
**10.27–11.18**	0.103358	9.67511
**12.30–13.13**	0.122398	8.17007
**13.97–14.50**	0.114104	8.76393
**Fast**	0.060405	16.55492

**Table 8:** Commonly used polymer properties.

Name of Polymer	Abbreviated Name	Repetition Unit	Density (g/cm^3^)
**Polyethylene**	PE	C_2_H_4_	0.88
**Polystyrene**	PS	C_8_H_8_	0.96
**Polyvinylchloride**	PVC	C_2_H_3_Cl	1.10
**Polymethylmethacrylate**	PMMA	C_3_H_8_O_2_	1.15
**Polyvinylalcoho**	PVA	C_2_H_4_O	1.19
**Polycarbonate**	PC	C_16_H_16_O_4_	1.24
**Polyethyleneterephthalat**	PET	C_10_H_8_O_4_	1.38
**Polytetrafluoroethylene**	PTFE	C_2_F_4_	2.20

**Table 9:** Gamma mass attenuation coefficients of different polymers compared to PSU.

Gamma Energy (MeV)	PSU	PE	PS	PVC	PMMA	PVA	PC	PET	PTFE
	Gamma Mass Attenuation Coefficients (cm^2^/g)								
**0.1**	0.162476	0.171692	0.162186	0.188806	0.167810	0.165530	0.160432	0.158313	0.149690
**0.2**	0.128660	0.139230	0.131315	0.129832	0.134994	0.133233	0.129294	0.127296	0.117980
**0.3**	0.111754	0.121303	0.114387	0.110589	0.117487	0.115963	0.112557	0.110784	0.102395
**0.4**	0.100394	0.109067	0.102845	0.098779	0.105604	0.104237	0.101182	0.099580	0.091963
**0.5**	0.091874	0.099846	0.094149	0.090179	0.096663	0.095413	0.092619	0.091150	0.084149
**0.6**	0.085079	0.092478	0.087201	0.083407	0.089525	0.088368	0.085781	0.084419	0.077922
**0.7**	0.079508	0.086431	0.081499	0.077888	0.083668	0.082587	0.080171	0.078897	0.072816
**0.8**	0.074862	0.081386	0.076742	0.073300	0.078782	0.077765	0.075490	0.074290	0.068559
**0.9**	0.070852	0.077031	0.072636	0.069350	0.074565	0.073602	0.071449	0.070313	0.064886
**1**	0.067192	0.073053	0.068885	0.065756	0.070715	0.069802	0.067760	0.066683	0.061535
**2**	0.046605	0.050573	0.047717	0.045844	0.049011	0.048380	0.046969	0.046240	0.042763
**3**	0.037280	0.040235	0.038028	0.037211	0.039121	0.038620	0.037502	0.036960	0.034392
**4**	0.031913	0.034207	0.032402	0.032430	0.033399	0.032976	0.032029	0.031611	0.029641
**5**	0.028413	0.030229	0.028703	0.029422	0.029650	0.029278	0.028446	0.028116	0.026583
**6**	0.025958	0.027407	0.026088	0.027387	0.027008	0.026673	0.025923	0.025661	0.024464
**7**	0.024146	0.025302	0.024143	0.025939	0.025050	0.024742	0.024053	0.023846	0.022919
**8**	0.022759	0.023674	0.022645	0.024874	0.023546	0.023259	0.022618	0.022456	0.021753
**9**	0.021669	0.022380	0.021457	0.024070	0.022357	0.022087	0.021484	0.021361	0.020848
**10**	0.020791	0.021328	0.020494	0.023450	0.021397	0.021141	0.020569	0.020478	0.020129
**15**	0.018196	0.018121	0.017585	0.021847	0.018521	0.018308	0.017832	0.017857	0.018084
**20**	0.016993	0.016538	0.016174	0.021338	0.017151	0.016960	0.016533	0.016629	0.017218

**Table 10:** Neutron effective removal cross-sections of different polymers compared to PSU.

Neutron Energy (MeV)	PSU	PE	PS	PVC	PMMA	PVA	PC	PET	PTFE
	Neutron Effective Removal Cross-Sections (cm^−1^)								
**0.1**	0.732690	1.132100	0.763980	0.515950	1.117500	1.035000	0.796740	0.699570	1.398800
**0.2**	0.580890	0.887670	0.615320	0.410770	0.880900	0.821740	0.645240	0.576560	0.331320
**0.3**	0.500440	0.747520	0.525770	0.348750	0.749060	0.701770	0.558350	0.504470	0.555260
**0.4**	0.472980	0.659400	0.466890	0.326050	0.723040	0.676320	0.536080	0.512120	0.512160
**0.5**	0.422520	0.599510	0.429170	0.299230	0.631020	0.592060	0.473950	0.443790	0.427180
**0.6**	0.378590	0.544210	0.391860	0.266240	0.550870	0.519480	0.420820	0.387460	0.452470
**0.7**	0.359140	0.509700	0.366840	0.256300	0.514660	0.485660	0.394600	0.361800	0.278750
**0.8**	0.333130	0.474890	0.342760	0.246770	0.481660	0.453330	0.368620	0.339460	0.294740
**0.9**	0.316980	0.442700	0.321330	0.227340	0.462260	0.435720	0.353230	0.331850	0.273410
**1**	0.338180	0.424450	0.307440	0.225400	0.532410	0.497920	0.392350	0.403710	0.238470
**2**	0.201280	0.283470	0.204550	0.160880	0.286590	0.269780	0.218660	0.200830	0.228850
**3**	0.153410	0.217660	0.154910	0.132780	0.221500	0.207450	0.165980	0.152120	0.145730
**4**	0.180380	0.219540	0.173170	0.136910	0.231030	0.222280	0.192890	0.187360	0.146560
**5**	0.127310	0.169930	0.126390	0.110650	0.171600	0.162620	0.135810	0.126600	0.117350
**6**	0.119620	0.150850	0.113380	0.099863	0.165490	0.156670	0.130020	0.126520	0.118160
**7**	0.091677	0.124580	0.089856	0.084969	0.129880	0.122160	0.098687	0.092388	0.103000
**8**	0.137440	0.157500	0.133850	0.102820	0.150370	0.149540	0.141540	0.135420	0.137920
**9**	0.102020	0.120290	0.096083	0.082091	0.127920	0.123620	0.108250	0.105990	0.122750
**10**	0.101280	0.115970	0.094039	0.079146	0.124980	0.121240	0.107170	0.106180	0.122020
**15**	0.103560	0.102840	0.091645	0.072025	0.118070	0.116800	0.109630	0.113740	0.131710
**20**	0.100750	0.093644	0.088715	0.068785	0.106200	0.107530	0.105740	0.110370	0.134850

**Table 11:** The fast neutron removal cross-sections of all investigated polymers.

Element	Weight Fraction	Σ*_R_*/*ρ* (cm^2^/g)	Partial Density(g/cm^3^)	Σ*_R_* (cm^−1^)
**PSU**				
**H (Hydrogen)**	0.05011	0.19000	0.06214	0.01181
**C (Carbon)**	0.73281	0.05019	0.90869	0.04560
**O (Oxygen)**	0.14462	0.04053	0.17933	0.00727
**S (Sulfur)**	0.07246	0.02610	0.08985	0.00234
				**0.06702**
**PE**				
**H (Hydrogen)**	0.14372	0.19000	0.12647	0.02403
**C (Carbon)**	0.85628	0.05019	0.75353	0.03782
				**0.06185**
**PS**				
**H (Hydrogen)**	0.07742	0.19000	0.07433	0.01412
**C (Carbon)**	0.92258	0.05019	0.88567	0.04445
				**0.05857**
**PVC**				
**H (Hydrogen)**	0.04838	0.19000	0.05322	0.01011
**C (Carbon)**	0.38435	0.05019	0.42279	0.02122
**Cl (Chlorine)**	0.56726	0.02522	0.62399	0.01574
				**0.04707**
**PMMA**				
**H (Hydrogen)**	0.10597	0.19000	0.12186	0.02315
**C (Carbon)**	0.47352	0.05019	0.54455	0.02733
**O (Oxygen)**	0.42051	0.04053	0.48359	0.01960
				**0.07008**
**PVA**				
**H (Hydrogen)**	0.09152	0.19000	0.10891	0.02069
**C (Carbon)**	0.54529	0.05019	0.64890	0.03257
**O (Oxygen)**	0.36319	0.04053	0.43219	0.01752
				**0.07077**
**PC**				
**H (Hydrogen)**	0.05923	0.19000	0.07344	0.01395
**C (Carbon)**	0.70575	0.05019	0.87512	0.04392
**O (Oxygen)**	0.23503	0.04053	0.29144	0.01181
				**0.06968**
**PET**				
**H (Hydrogen)**	0.04196	0.19000	0.05791	0.01100
**C (Carbon)**	0.62501	0.05019	0.86252	0.04329
**O (Oxygen)**	0.33303	0.04053	0.45958	0.01863
				**0.07291**
**PTFE**				
**C (Carbon)**	0.24018	0.05019	0.52839	0.02652
**F (Fluorine)**	0.75982	0.03612	1.67161	0.06038
				**0.08690**

## Data Availability

The data presented in this study are available on request from the corresponding author.

## References

[1] Singh T., Rajni, Kaur U., Singh P.S. (2010). Photon energy absorption parameters for some polymers. Ann. Nucl. Energy.

[2] Mann K.S., Rani A., Heer M.S. (2015). Shielding behaviors of some polymer and plastic materials for gamma-rays. Radiat. Phys. Chem..

[3] Al-Buriahi M.S., Eke C., Alomairy S., Yildirim A., Alsaeedy H.I., Sriwunkum C. (2021). Radiation attenuation properties of some commercial polymers for advanced shielding applications at low energies. Polym. Adv. Technol..

[4] Sayyed M. (2016). Investigation of shielding parameters for smart polymers. Chin. J. Phys..

[5] Akhdar H., Marashdeh M., AlAqeel M. (2022). Investigation of gamma radiation shielding properties of polyethylene glycol in the energy range from 8.67 to 23.19 keV. Nucl. Eng. Technol..

[6] Tuna T., Eker A.A., Kam E. (2021). Neutron shielding characteristics of polymer composites with boron carbide. J. Korean Phys. Soc..

[7] Korkut T., Gencel O., Kam E., Brostow W. (2013). X-ray, Gamma, and Neutron Radiation Tests on Epoxy-Ferrochromium Slag Composites by Experiments and Monte Carlo Simulations. Int. J. Polym. Anal. Charact..

[8] Aygün B., Korkut T., Karabulut A., Gencel O., Karabulut A. (2015). Production and Neutron Irradiation Tests on a New Epoxy/Molybdenum Composite. Int. J. Polym. Anal. Charact..

[9] Sharma A., Singh B., Sandhu B. (2019). Investigation of photon interaction parameters of polymeric materials using Monte Carlo simulation. Chin. J. Phys..

[10] Physical, Mechanical, Thermal, Electrical, and Optical Properties of PSU. https://www.curbellplastics.com/Research-Solutions/Plastic-Material-Properties/PSU-Properties.

[11] Foster Corporation Polysulfones in Healthcare Applications—Foster Corporation. https://www.fostercomp.com/polysulfones-in-healthcare-applications.

[12] Microspec Corporation (2022). Polysulfone|Microspec Corporation. https://www.microspecorporation.com/materials/engineering-resins/polysulfone.

[13] Dielectric Manufacturing (2022). Material Properties of Thermoplastic Polysulfone–PSU. https://dielectricmfg.com/knowledge-base/psu-polysulfone.

[14] Sterling Plastics Inc.-Blaine MN Plastics Distributor POLYSULFONE (PSU). http://sterlingplasticsinc.com/materials/polysulfone-psu..

[15] Hubbell J. (1982). Photon mass attenuation and energy-absorption coefficients. Int. J. Appl. Radiat. Isot..

[16] Kaewkhao J., Laopaiboon J., Chewpraditkul W. (2008). Determination of effective atomic numbers and effective electron densities for Cu/Zn alloy. J. Quant. Spectrosc. Radiat. Transf..

[17] Un A., Demir F. (2013). Determination of mass attenuation coefficients, effective atomic numbers and effective electron numbers for heavy-weight and normal-weight concretes. Appl. Radiat. Isot..

[18] Singh K., Singh H., Sharma V., Nathuram R., Khanna A., Kumar R., Bhatti S.S., Sahota H.S. (2002). Gamma-ray attenuation coefficients in bismuth borate glasses. Nucl. Instrum. Methods Phys. Res. Sect. B Beam Interact. Mater. Atoms..

[19] Olukotun S., Mann K.S., Gbenu S., Ibitoye F., Oladejo O., Joshi A., Tekin H., Sayyed M., Fasasi M., Balogun F. (2019). Neutron-shielding behaviour investigations of some clay-materials. Nucl. Eng. Technol..

[20] Singh Mann K. (2015). Toolkit for Fast Neutron Removal Cross-Section. Proceedings of the 3rd International Conference Advancements in Engineering and Technology.

[21] El Abd A., Mesbah G., Mohammed N.M.A., Ellithi A. (2017). A simple Method for Determining the Effective Removal Cross Section for Fast Neutrons. J. Radiat. Nucl. Appl..

[22] Agostinelli S., Allison J., Amako K., Apostolakis J., Araujo H., Arce P., Asai M., Axen D., Banerjee S., Barrand G. (2003). Geant4—A simulation toolkit. Nucl. Instrum. Methods Phys. Res. Sect. A Accel. Spectrometers Detect. Assoc. Equip..

[23] Brun R., Rademakers F. (1997). ROOT—An object oriented data analysis framework. Nucl. Instrum. Methods Phys. Res. Sect. A Accel. Spectrometers Detect. Assoc. Equip..

[24] Hila F.C., Asuncion-Astronomo A., Dingle C.A.M., Jecong J.F.M., Javier-Hila A.M.V., Gili M.B.Z., Balderas C.V., Lopez G.E.P., Guillermo N.R.D., Amorsolo A.V. (2021). EpiXS: A Windows-based program for photon attenuation, dosimetry and shielding based on EPICS2017 (ENDF/B-VIII) and EPDL97 (ENDF/B-VI.8). Radiat. Phys. Chem..

[25] Mirji R., Lobo B. (2017). Computation of the mass attenuation coefficient of polymeric materials at specific gamma photon energies. Radiat. Phys. Chem..

[26] Hehn G. (1986). Principles of Radiation Shielding. Nucl. Technol..

[27] El-Khayatt A. (2010). Calculation of fast neutron removal cross-sections for some compounds and materials. Ann. Nucl. Energy.

[28] Elwahab N.R.A., Helal N., Mohamed T., Shahin F., Ali F.M. (2019). New shielding composite paste for mixed fields of fast neutrons and gamma rays. Mater. Chem. Phys..

